# Diet Quality and Eating Frequency Were Associated with Insulin-Taking Status among Adults

**DOI:** 10.3390/nu16203441

**Published:** 2024-10-11

**Authors:** Luotao Lin, Yue Qin, Emily Hutchins, Alexandra E. Cowan-Pyle, Jiangpeng He, Fengqing Zhu, Edward J. Delp, Heather A. Eicher-Miller

**Affiliations:** 1 Department of Individual, Family, and Community Education, University of New Mexico, Albuquerque, NM 87131, USA; llin@unm.edu; 2 Department of Nutrition Science, Purdue University, West Lafayette, IN 47907, USA; qin53@purdue.edu (Y.Q.); hutchi45@purdue.edu (E.H.); 3 Institute for Advancing Health through Agriculture, Texas A&M University, College Station, TX 77840, USA; alexandra.cowan@ag.tamu.edu; 4 School of Electrical and Computer Engineering, Purdue University, West Lafayette, IN 47907, USA; he416@purdue.edu (J.H.); zhu0@purdue.edu (F.Z.); ace@ecn.purdue.edu (E.J.D.)

**Keywords:** insulin, diet quality, eating frequency, adults, diabetes, eating time

## Abstract

Objective: This pilot cross-sectional study explored differences in dietary intake and eating behaviors between healthy adults and a group of adults taking insulin to manage diabetes. Methods: A characteristic questionnaire and up to four Automated Self-Administered 24-Hour dietary recalls were collected from 152 adults aged 18–65 years (96 healthy and 56 adults taking insulin) from Indiana and across the U.S. from 2022 to 2023. The macronutrient intake, diet quality via the Healthy Eating Index (HEI)-2015, eating frequency, and consistency of timing of eating were calculated and compared between the two groups using adjusted linear or logistic regression models. Results: The total mean HEI scores were very low, at 56 out of 100 and 49 out of 100 for the healthy and insulin-taking groups, respectively. Insulin-taking adults had significantly lower HEI total (*p =* 0.003) and component scores compared to the healthy group for greens and beans (2.0 vs. 3.0, *p =* 0.02), whole fruit (2.1 vs. 2.9, *p =* 0.05), seafood and plant proteins (2.1 vs. 3.3, *p =* 0.004), and saturated fats (3.7 vs. 5.4, *p =* 0.05). Eating frequency was significantly lower in the insulin-taking group than in the healthy group (3.0 vs. 3.4 eating occasions/day, *p =* 0.05). Conclusion: Evidence of the low diet quality and eating frequency of insulin takers may help inform and justify nutrition education to control and manage diabetes.

## 1. Introduction

Approximately 11% of the U.S. population (37.3 million) has diabetes, the eighth leading cause of death in the U.S. [1,2]. About 8.4 million adults take insulin to treat their diabetes [3]. Dietary interventions and lifestyle behavior modifications play critical roles in the management of diabetes and could reduce the need for medication and insulin therapies in certain scenarios for patients with diabetes [4,5]. Since diet and lifestyle are related to the prevention of other health outcomes and wellness, the implementation of dietary and lifestyle interventions could improve diabetes management, increase the quality of life for patients, and potentially lower medical costs. In addition to carbohydrates, inclusion of other macronutrients in meal composition has the potential to impact glycemic control and diabetes management, such as fat and protein [6]. Greater dietary quality has been shown to be significantly associated with more optimal glycemic control among patients with Type 1 [7] or Type 2 diabetes [8] and a lower incidence of diabetes [9]. The Academy of Nutrition and Dietetics Diabetes Type 1 and 2 (2015) Evidence-Based Nutrition Practice Guidelines emphasized the importance of consistent carbohydrate intake across meals, with respect to the timing of intake and amount of carbohydrates consumed [10]. Eating frequency is another important factor for diabetic management. Previous studies have shown that spreading intake throughout the day with smaller, more frequent eating occasions can potentially lower the glycemic load, and higher eating frequency may bring more optimal diabetic control. A higher eating frequency may also lead to smaller meals and reduced stomach distension, which may cause a slower rate of stomach emptying, nutrient delivery, and less insulin required to control blood glycemic levels [11,12].

Despite this prior evidence and recommendation, there is a lack of information on overall dietary intake, macronutrient composition, diet quality, eating frequency, and consistency of the timing of eating occasions throughout a day among patients taking insulin. Two studies described the dietary quality of those classified with diabetes compared with healthy individuals [13,14], and none focused on individuals that use insulin. The urgency to control and manage diabetes, and the critical roles those multiple aspects of diet (diet quality, macronutrient composition, eating frequency, etc.) play in diabetes management as well as in insulin use, justify a need to examine these aspects of insulin takers’ diets. The findings may be used to inform diet-related interventions and policy recommendations to further improve treatment and quality of life for those using insulin. Further, comparing insulin takers’ diets to those of a group not using insulin (i.e., a healthy group) may help differentiate eating patterns to better understand how the diets of insulin takers may vary from those of the general population. Therefore, the objective of this pilot study was to assess and compare the dietary intake, specifically, the energy and macronutrient composition, dietary quality, eating frequency, and consistency (between two days) of the timing of eating occasions, between a group of adults with diabetes that use insulin and a group that were healthy. It was hypothesized that dietary quality and eating frequency would be higher, energy and carbohydrate intake would be lower, and the timing of meals throughout two 24 h days would be more consistent in the insulin-taking group compared with the healthy group in a convenience sample of those taking insulin and a healthy group of adults from Indiana and across the U.S. aged 18–65 years.

## 2. Methods

### 2.1. Study Design and Sample

**Figure 1:**
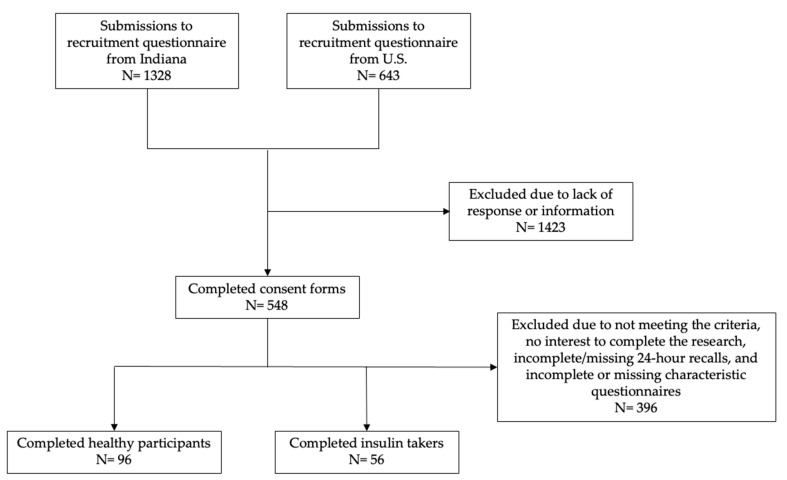
Flow chart of participants in this pilot study.

Both insulin-taking individuals and those considered “healthy” (i.e., not diagnosed with a health condition that required diet modifications) were recruited in-person from Indiana and online across the U.S. from February 2022 to April 2023, using a cross-sectional study design. Physical and digital flyers were posted in several places to recruit participants for this convenience sample: physical flyers on the campus of Purdue University, community and health centers in West Lafayette, IN and Lafayette, IN, the Eskenazi Health hospital in Indianapolis, IN, and through Purdue Extension at the Indiana county and state fairs; digital flyers were posted on Purdue University’s research-posting website, the Purdue Extension program “Dining with Diabetes” website, the Indiana Clinical and Translational Science Institute’s study-posting platform iConnect, and through social media advertisements (Facebook and Instagram) to recruit more participants across the country. All potential participants answered the recruitment questionnaires to indicate their interest of participating in this pilot study and provided signed informed consent. Participants completed all steps of the study online, including a screener to identify the inclusion criteria: those aged 18 to 65 years with access to the use of a smart phone to record dietary recalls. Insulin takers were self-identified as those using insulin and the participants considered “healthy” were self-identified as those not diagnosed with a health condition that required dietary modifications. Patients completed a brief characteristics questionnaire online through Qualtrics and up to four 24 h dietary recalls using the Automated Self-Administered 24-Hour Dietary Recall (ASA-24). Participants were compensated $40 in Amazon gift cards if they successfully completed two or more dietary recalls, and $20 if they only completed one 24 h dietary recall and the characteristics questionnaire. This analysis included 152 participants (flow chart in Figure 1) distributed as 96 healthy participants and 56 insulin-taking participants. Participants were excluded from this pilot study if they did not meet the study criteria, had no interest in completing the research, had not completed the 24 h dietary recall, and/or had incomplete or missing characteristic questionnaire data. Among those who completed the study, 122 reported two or more 24 h dietary recalls (80% of the sample); 83 of these were healthy and 39 were insulin taking. There were 13 in the healthy group and 17 in the insulin-taking group who had only completed one 24 h dietary recall. Participants were asked to complete the dietary recalls on one weekday (i.e., Monday–Friday) and one weekend day (i.e., Saturday or Sunday), and 58 (38% of the sample) completed their recalls on a week and weekend day; of those, 38 were healthy and 20 were insulin taking. In the healthy group, 45 participants only had weekday dietary recalls, and 13 participants only had weekend day dietary recalls. In the insulin-taking group, 30 participants only had weekday dietary recalls, and five participants only had weekend day dietary recalls. Purdue University’s Institutional Review Board approved (IRB #: IRB-2021-1734) all study protocols and participants signed the informed consent forms before beginning study procedures.

### 2.2. Sociodemographic Characteristics

Self-reported sociodemographic characteristics included sex (male or female), age (18–30 or 31–65 years old), race/ethnicity (American Indian or Alaska Native, Asian, Black/African American, Hispanic/Latino, White, or two or more races selected), level of education attainment (high school/some college/Associate’s degree, Bachelor’s degree, Master’s degree, or Doctoral/professional degree), marital status (married or widowed/divorced/separated/never married/single), income level (<USD 20,000 or >USD 20,000), and employment status (employed or unemployed). All characteristics were included in the model as categorical covariates due to the nature of the variables and the uneven distribution of variables including age, race/ethnicity, and marital status.

### 2.3. Dietary Assessment

The ASA-24 dietary recalls queried all foods and beverages consumed in the past 24 h (i.e., from midnight to midnight). Participants were also asked about the timing, frequency, and category of eating occasions, as well as the sources of the various foods and beverages they reported. Each reported food item was linked to the U.S. Department of Agriculture’s Food and Nutrient Database for Dietary Studies (2019–2020) [15] and FoodData Central [16] to calculate the respective energy and macronutrient intake values. Dietary information was also linked to the Food Pattern Equivalents Database (FPED) (2017–2018) [17] to disaggregate foods and their amounts into dietary components (e.g., added sugar) for the estimation of the dietary quality scores. Dietary quality was approximated using the Healthy Eating Index (HEI)-2015, the current version of the HEI at study initiation. The HEI-2015 is a density-based score that quantifies dietary adherence to the 2015–2020 Dietary Guidelines for Americans (DGA) [18]. The HEI-2015 total score is the sum of 13 dietary components for adequacy, including total fruit, whole fruit, total vegetables, greens and beans, whole grains, dairy, total protein foods, seafood and plant proteins, and fatty acids; dietary components for moderation, such as saturated fats, refined grains, sodium, and added sugars, are reverse scored [19]. A higher HEI-2015 total score (maximum score of 100) reflects closer alignment to the 2015–2020 DGA [20]. The HEI-2015 scores were calculated using the simple HEI scoring algorithm at the individual level, using all completed recalls, which construed ratios for each individual per 1000 kcal of energy [21]. For every 24 h dietary recall completed by a participant, each dietary intake component was summed and divided by the sum of the total energy to calculate the ratio, which was then compared with the applicable scoring standards.

### 2.4. Eating Frequency and Consistency of Timing in Eating Occasions

Meal frequency was determined by calculating the average of the total number of meal occasions participants reported, including breakfast, brunch, lunch, supper, and dinner. Snack frequency was calculated as the average of the total number of snack occasions participants reported. Eating frequency was calculated as the sum of the average meal frequency and snack frequency per day, resulting in the total number of eating occasions of the participant per day.

Consistency of timing in eating occasions was calculated based on the differences in timing of eating between two days of dietary recalls from 122 participants who had at least two dietary recalls. For 12 participants who had more than two dietary recalls, only two random dietary recalls were selected to calculate the time difference of eating meals. Nearly half (58 participants) had one weekday and one weekend day represented in their dietary recalls. As described above, the timing of each eating occasion was recorded through the ASA-24 for both days of recalls. Eating occasions were also self-classified as meals (breakfast, brunch, lunch, dinner, and supper) and as snacks. The same types of meals between the two dietary recalls were matched to directly calculate the time differences between similar meals on the two different days. For example, if two matched meals such as “breakfast” occurred at 9:00 a.m. on the first recall and 10:00 a.m. on the second recall, the time difference was 60 min and represented a 60 min inconsistency in the timing of the breakfast eating occasions. Unmatched meals were assigned a time penalty, which was calculated by (24 h–8 h) × 60 min/h/3 = 320 min. For example, one participant had two meals in the first dietary recall (a breakfast at 7:00 a.m. and a dinner at 8:00 p.m.), and three meals in the second dietary recall (a breakfast at 6:00 a.m., a lunch at 2:00 p.m., and a dinner at 8:00 p.m.). The lunch on the second dietary recall was the unmatched meal, and therefore, the participant received a time penalty of 320 min. Total time difference between meals of two days of dietary recalls was also calculated as the total of the time differences of matched meals and unmatched meals’ time penalties.

Similarly, consistency of timing of snacking was evaluated based on the time differences of snacking events (including just a drink) between the two days of dietary recalls from 78 participants who had at least two dietary recalls and reported at least one snacking event in the two dietary recalls. Snacking events between the two dietary recalls were matched if the time difference was less than 320 min; unmatched snacking occasions received a 320 min penalty. Therefore, the total time difference between the snacking occasions of two dietary recalls was calculated as the sum of time differences of matched snack and unmatched snack time penalties.

Total timing differences of eating occasions was the sum of the time differences of meals and snacks between the two dietary recalls of each participant.

### 2.5. Statistical Analysis

Chi-square tests were used to compare sociodemographic characteristics between healthy participants and insulin takers to understand the profile of the groups and to identify statistically significant differences between groups. Based on that analysis, the multiple linear regression was adjusted by age group, race/ethnicity in two categories (Non-Hispanic White and Others), marital status, and student status. Pearson correlation coefficients, tolerances, variance inflations, and eigenvalues were tested to check for potential multicollinearity among age group, marital status, and student status. The variables had low correlation (all Pearson correlation coefficients < 0.8), tolerances were all larger than 0.1, variance inflations were all less than 10, and all eigenvalues were not closer to 0, all indicating lack of multicollinearity among age group, marital status, and student status. Multiple linear regression was used to investigate any potential differences in the outcomes of energy and macronutrient intake, total and component HEI-2015 scores, and consistency in the timing of eating occasions between groups. Statistical power to detect statistically significant differences in HEI scores between the groups (effect size of 0.74, power of 80%) was confirmed from a previous study comparing dietary quality between healthy individuals and insulin takers [13]. Statistical significance was set at *p* ≤ 0.05.

## 3. Results

Of the 152 participants in this pilot study, 56 were in the insulin taking group and 96 participants were in the healthy group. Sociodemographic characteristics are presented in Table 1; statistically significant differences were observed by age group, race/ethnicity group, marital status, and student status between the insulin taking and healthy groups. The insulin-taking group had a higher percentage of participants in the 31–65 age group, non-Hispanic white group, married category, and non-student group; meanwhile, the healthy group had a higher percentage of participants in the 18–30 age group, Asian group, unmarried category, and student group (all *p <* 0.05).

The mean daily macronutrient and energy intakes among the insulin-taking and healthy groups are presented in Table 2. On average, participants in the healthy group consumed 1688.4 kcal of energy with 46% of energy from carbohydrate, 17% from protein, and 37% from fat; meanwhile, participants in the insulin taking group, on average, consumed 1684.3 kcal of energy with 41% of energy originating from carbohydrate, 19% from protein, and 40% from fat. Significant differences between the groups were not detected.

Dietary quality, using the HEI-2015, was assessed for both the healthy and insulin taking groups (Table 3). Participants in the insulin taking group had significantly lower mean HEI-2015 scores compared with the healthy group, including total scores (48.8 vs. 56.4, *p =* 0.003), and component scores for greens and beans (2.0 vs. 3.0, *p =* 0.02), whole fruit (2.1 vs. 2.9, *p =* 0.05), seafood and plant proteins (2.1 vs. 3.3, *p =* 0.004), and saturated fats (3.7 vs. 5.4, *p =* 0.05).

**Figure 2:**
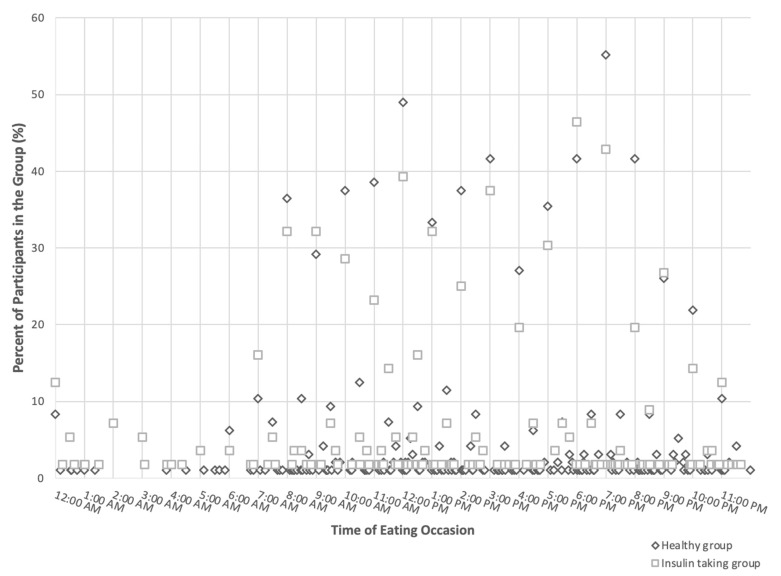
Distribution of timing of eating in an insulin-taking and healthy group within a convenience sample of Indiana and U.S. adults aged 18–65 years (n = 152).

The time of the eating occasions of the participants in the two groups is shown in Figure 2. Each dot represents the non-zero percentage of participants in the group (y-axis) that had eating occasions at the specific time in the 24 h day shown in hourly increments on the x-axis. Overall, most of the eating occasions occurred during 8 a.m. to 9 p.m.; a smaller proportion of the participants in the insulin-taking group (square) had eating occasions during this time compared to those in the healthy group (rhombus). More than 50% and 55% of the participants in the healthy group had an eating occasion at 12 p.m. and 7 p.m., respectively. Almost 40% of the participants in the insulin-taking group had an eating occasion at 12 p.m., and more than 40% of the participants in the insulin-taking group had an eating occasion at 6 p.m. or 7 p.m.

The insulin-taking group had a significantly lower eating frequency than the healthy group, despite that no significant differences between the two groups for meal and snack frequency were observed (Table 4). The consistency in eating occasions was statistically compared using the calculated mean time differences of eating occasion, meals, and snacks during the two days of dietary recalls in Table 4. No significant differences in consistency were observed between the two groups.

## 4. Discussion

In this pilot study, dietary intake, diet quality, eating frequency, and the consistency of the timing of eating occasions of participants taking insulin were investigated and compared with those of healthy participants. The results suggested that participants taking insulin have a significantly lower diet quality than the healthy group, mainly due to a lower intake of greens, whole fruits, and beans, as well as seafood and plant proteins, and higher intake of saturated fats. The insulin-taking group also had a significantly lower eating frequency than the healthy group, despite that no significant differences between the two groups for meal and snack frequency were observed. Neither energy and macronutrient intakes nor the consistency in timing of eating occasions differed between the insulin taking and healthy groups.

Diet plays a critical role in the management of diabetes among those who are taking insulin. In this pilot study, mean HEI-2015 scores were at 56 out of 100 for the healthy group and only 49 out of 100 for the insulin-taking group; both scores were lower than that of the general U.S. adult population (HEI-2015 total score: 57 out of 100) [22]. While it was hypothesized that those in the insulin-taking group may be prioritizing dietary quality to manage diabetes and, thus, exhibit higher HEI-2015 scores, the insulin-taking group had an even lower dietary quality than the healthy group in this pilot study. The difference in total HEI-2015 scores between the insulin-taking group and the healthy group (β = −7.5 ± 2.4, *p =* 0.003) was not only statistically significant but also meaningful to health because the difference was large enough to reduce the risk of abdominal obesity [23]. Diet quality has been significantly associated with blood glucose regulation, treatment, and control for diabetic patients [24,25,26]. Compared to their counterparts, participants in the insulin taking group may increase intake of greens and beans, whole fruit, seafood and plant proteins and decrease the intake of saturated fats to improve dietary quality [27]. The difference in saturated fats between the two groups was particularly noteworthy. This result indicated the abundance of saturated fats in the US diet [28]. Since carbohydrate restriction has been shown to be beneficial to diabetic management [29,30], this dietary practice has been widely recommended to diabetic patients and, especially, those who are using insulin for better diabetic control and to reduce postprandial glucose excursion. Those taking insulin, in the results here, may have potentially responded to this advice by lowering carbohydrates and increasing saturated fat intake, yielding the lower saturated fat component scores (as they are reverse scored) that add to the lower total HEI scores [31,32]. This practice of restricting carbohydrates may potentially further contribute to the low HEI score because carbohydrates are found in foods contributing to the component scores of the total HEI score and that were differential among the insulin-taking and healthy groups here, such as greens and beans and whole fruits. The small but significantly lower consumption of whole fruits among insulin takers compared to the healthy group is also notable because previous studies showed that whole fruits consumption had an inverse relationship with the risk of developing Type 2 diabetes and may protect against the incidence of Type 2 diabetes [31,32,33] so, although these foods are rich in carbohydrates, they may be a helpful component of the diet for those with diabetes. Also of note, the finding of low diet quality among the insulin-taking group was not consistent with that of a previous study in Denmark [34] where patients with Type 1 and Type 2 diabetes had higher adherence to dietary recommendations than the general population. Another study showed that there are multiple challenges for diabetic patients and, especially, those using insulin to adhere to healthy diets, such as financial constraints, unsupportive social and physical environments, and personal factors [35]. The access to a healthful dietary environment where foods supporting dietary quality are accessible throughout the day and nutrition education to make healthful dietary choices is provided may be important for both groups to improve dietary quality. Furthermore, nutrition education provided through primary care providers or dieticians may potentially help patients taking insulin to improve dietary quality and support diabetes management.

Macronutrient intake, especially carbohydrate intake, is important for patients with diabetes for managing blood glucose level. Carbohydrate intake is directly linked to glucose response and proper insulin dosing. The American Diabetes Association guidelines [36] stated that macronutrient distribution should be based on an individual assessment of current eating patterns, preferences, and metabolic goals but that a starting point may be 45% of energy intake from carbohydrate [37]. Therefore, despite that the carbohydrate intake in the insulin-taking group was 20 g lower, but not significantly so, compared with the healthy group, carbohydrate intake may comply better with guidance if increased.

For patients on fixed insulin, not only is the amount of carbohydrate intake important, but the consistent timing of eating occasions is too [10]. The insulin-taking group had a significantly lower eating frequency of all occasions than the healthy group, yet there were not significant findings in the separate categories of meals and snacks. The results suggested that participants taking insulin had fewer average meals and snacks together per day. One potential explanation may be that those using insulin want to lessen the amount of dosing they need after each eating event, which could mean a lower eating frequency [38]. Although a lower frequency may not necessarily be unhealthful, a previous study has identified disordered eating behaviors among those using insulin, suggesting attempts to limit dosing [39]. Insulin takers may also wish to minimize the number of insulin injections due to many other issues [40], and in doing so, they reduce the eating frequency. Inconsistent and irregular eating time is not favorable to controlling blood glucose levels [6,41,42]. Previous studies also suggested that meal frequency was helpful, with significantly negative associations with hemoglobin A1C [42] and a lower risk for diabetes [43]. Another factor in the consistency in the timing and intake of meals in this study might have been the evaluation of weekdays and weekend days. The consistency in the timing and intake of meals during the week is much higher than that of weekday vs. weekend days [44]. This study included both types of days in the evaluation of consistency, but many participants (47.4%) did have two weekdays. Yet, if all participants had two weekdays, the results may have shown more convergence in consistency. Nutrition education and learning to build healthy eating habits may be important for those using insulin, especially to maintain a consistent carbohydrate or dietary intake with respect to time and amount to potentially inform improved blood glucose control.

One of the strengths of this pilot study was that it contributed to the literature on the diet of insulin takers, which provides information on diet among insulin takers and evidence to support blood glucose management. The limitations of this pilot study were that the study had a relatively small sample and unbalanced groups. Due to the difficulties in recruiting individuals taking insulin, the study only included 56 insulin takers, which was a smaller group (n = 56) than the healthy group (n = 96). However, according to the G*Power 3.1, the power of the current study was 0.91, which is acceptable. A larger and more balanced sample may have yielded more opportunity to observe significant differences between the two groups. In addition, the unbalanced sociodemographic characteristics, especially in age, between the two groups could also affect the results. Also, the severity, length, and type of diabetes among participants were not collected in this study. Diets of those taking insulin may vary significantly and the study represents an unknown mix of those with Type 1 and 2 diabetes. Additionally, whether the participants were currently receiving or had received nutrition counseling was not assessed, and could potentially cause differences in dietary intake between the two groups, as could the presence of other health conditions, such as obesity, kidney disease, and metabolic syndrome, that were not queried in the study. Furthermore, participants were mainly recruited from Indiana and the sample representation was largely non-Hispanic white, so generalization of the current results to a broader population is limited. Lastly, due to the nature of the cross-sectional study design, no causal relationships could be determined in this study and the insulin-taking status or healthy categorization may not be related to the differences between the two groups.

## 5. Conclusions

This pilot study suggests that diet quality was poor among both insulin-taking and healthy groups and was significantly associated with insulin-taking status. The insulin-taking group also had a significantly lower eating frequency than the healthy group. Evidence of a low diet quality and eating frequency among insulin takers may help to inform and justify nutrition education to control and manage diabetes and to tailor consumer education. This pilot study also explored the difference in energy and macronutrient intake and consistency of the timing of eating occasions between an insulin-taking group and a healthy group, which highlights the critical need to investigate different aspects of dietary behavior to tailor diabetes care and provide patients with comprehensive suggestions, leading to improved long-term diabetes outcomes.

## Figures and Tables

**Table 1:** Sociodemographic characteristics and comparisons between an insulin-taking and healthy group in a convenience sample of Indiana and U.S. adults aged 18–65 years (n = 152).

Characteristics	Total (n)	Healthy Group ^1^	Insulin-Taking Group ^1^	*p*-Value ^2^
	152	96 (63.2%)	56 (36.8%)	
Sex ^3^				0.38
Female	104	63 (66.3%)	41 (73.2%)	
Male	47	32 (33.7%)	15 (26.8%)	
Age group (years)				<0.0001 *
18–30	87	67 (69.8%)	20 (35.7%)	
31–65	65	29 (30.2%)	36 (64.3%)	
Race/ethnicity 4 categories ^3^				0.001 *
American Indiana/Alaska Native, Hispanic/Latino, and 2 or more groups	12	7 (7.6%)	5 (9.0%)	
Asian	34	31 (33.3%)	3(5.4%)	
Non-Hispanic Black/African American	12	6 (6.5%)	6 (10.7%)	
Non-Hispanic White	91	49 (52.7%)	42 (75.0%)	
Race/ethnicity 2 categories ^3,4^				0.007 *
Non-Hispanic White	91	49 (52.7%)	42 (75.0%)	
Others	58	44 (47.3%)	14 (25.0%)	
Education Level ^3^				0.11
High School or some college or Associate’s degree	30	15 (15.8%)	15 (26.8%)	
Bachelor’s degree	59	35 (36.8%)	24 (42.9%)	
Master’s degree	33	22 (23.2%)	11 (19.6%)	
Doctoral/professional degree	29	23 (24.2%)	6 (10.7%)	
Marital Status ^3^				0.04 *
Married	56	29 (31.2%)	27 (48.2%)	
Widowed/Divorced/Separated/Never married/Single	93	64 (68.8%)	29(51.8%)	
Income Level ^3^				0.18
≤USD 20,000	43	30 (35.3%)	13 (24.5%)	
>USD 20,000	95	55 (64.7%)	40 (75.5%)	
Employment Status				0.43
Employed (part-time/full-time)	97	59 (61.5%)	38 (67.9%)	
Unemployed or Other	55	37 (38.5%)	18 (32.1%)	
Student Status				0.0003 *
Student	58	47 (49.0%)	11 (19.6%)	
Non-student or Other	94	49 (51.0%)	45 (80.4%)	

^1^ Values are n (%). Total percentage may not be equal to 100% due to rounding. ^2^ χ^2^ *p*-value is a goodness-of-fit, one-sided test; * statistical significance is indicated when *p*-Value ≤ 0.05. ^3^ Missing data was due to participants choosing “prefer not to say”. ^4^ Race/ethnicity 2 groups was used in the final analysis.

**Table 2:** Comparison of macronutrient and energy intake between an insulin-taking and healthy group in a convenience sample of Indiana and U.S. adults aged 18–65 years (n = 152) ^1^.

Macronutrients	Healthy Group (n = 96)	Insulin-Taking Group (n = 56)	*p*-Value
Carbohydrates (g)	192.7 ± 78.0	173.8 ± 98.4	0.37
Fats (g)	70.3 ± 31.8	74.6 ± 35.1	0.99
Proteins (g)	74.6 ± 36.2	79.0 ± 43.9	0.70
Energy (kcal)	1688.4 ± 590.8	1684.3 ± 778.5	0.77

^1^ Multiple linear regression was adjusted by age group, race/ethnicity in 2 groups, marriage status, and student status. Values are shown as mean ± standard deviation. Statistical significance level is *p*-Value ≤ 0.05.

**Table 3:** Comparison of Healthy Eating Index-2015 total and component scores between an insulin taking and healthy group in a convenience sample of Indiana and U.S. adults ages 18–65 years (n = 152) ^1^.

HEI Components	Healthy Group (n = 96)	Insulin-Taking Group (n = 56)	Parameter Estimate ± Standard Error	*p*-Value
Total Score	56.4	48.8	−7.5 ± 2.4	0.003 *
Total Vegetables	3.6	3.5	−0.1 ± 0.3	0.63
Greens and Beans	3.0	2.0	−0.9 ± 0.4	0.02 *
Total Fruit	2.4	1.8	−0.5 ± 0.4	0.13
Whole Fruit	2.9	2.1	−0.8 ± 0.4	0.05 *
Whole Grains	3.1	2.1	−0.8 ± 0.6	0.18
Refined Grains	5.7	6.5	0.2 ± 0.6	0.72
Dairy	5.6	5.4	−0.5 ± 0.6	0.35
Total Protein Foods	4.3	4.4	0.1 ± 0.2	0.82
Seafood and Plant Proteins	3.3	2.1	−1.1 ± 0.4	0.004 *
Fatty Acids	5.4	4.3	−1.1 ± 0.6	0.10
Saturated Fats	5.4	3.7	−1.3 ± 0.6	0.05 *
Sodium	3.0	2.6	−0.6 ± 0.6	0.27
Added Sugars	8.5	8.2	−0.0 ± 0.4	0.96

^1^ Multiple linear regression was adjusted by age group, race/ethnicity 2 groups, marriage status, and student status. * Statistical significance is indicated when *p*-Value ≤ 0.05.

**Table 4:** Comparison of meal frequency (n = 152) and consistency of timing (n = 122) between an insulin-taking and healthy group in a convenience sample of Indiana and U.S. adults aged 18–65 years ^1^.

Frequency	Healthy Group (n = 96) ^2^	Insulin-Taking Group (n = 56) ^2^	*p*-Value
Eating frequency	3.4 ± 1.2	3.0 ± 1.1	0.05 *
Meal frequency	2.6 ± 0.7	2.4 ± 0.7	0.08
Snack frequency	0.8 ± 0.9	0.6 ± 0.7	0.41
**Consistency**	**Healthy Group (n = 83) ^2^**	**Insulin-Taking Group (n = 39) ^2^**	***p*-Value**
Time differences of all eating (minutes)	755.5 ± 378.6	700.6 ± 335.8	0.31
Time differences of meals (minutes)	308.1 ± 215.7	331.8 ± 265.2	0.60
Time differences of snacks	374.4 ± 242.3	410.9 ± 206.1	0.52

^1^ Multiple linear regression was adjusted by age group, race/ethnicity 2 groups, marriage status, and student status. ^2^ Values are shown as mean ± standard deviation. * Statistical significance level is *p*-Value ≤ 0.05.

## Data Availability

Data are unavailable due to privacy and ethical restrictions.
